# Genome editing of human embryos for research purposes: Japanese lay and expert attitudes

**DOI:** 10.3389/fgene.2023.1205067

**Published:** 2023-06-22

**Authors:** Kyoko Akatsuka, Taichi Hatta, Tsutomu Sawai, Misao Fujita

**Affiliations:** ^1^ Uehiro Research Division for iPS Cell Ethics, Center for iPS Cell Research and Application, Kyoto University, Kyoto, Japan; ^2^ Shizuoka Graduate University of Public Health, Shizuoka, Japan; ^3^ Graduate School of Humanities and Social Sciences, Hiroshima University, Higashi-Hiroshima, Japan; ^4^ Institute for the Advanced Study of Human Biology (ASHBi), Kyoto University, Kyoto, Japan

**Keywords:** CRISPR-Cas9, public survey, human genome editing, human embryo, biomedical research, acceptance, ethics, Japan

## Abstract

**Background:** Multiple surveys of the general public and experts on human genome editing have been conducted. However, many focused only on editing in clinical applications, with few regarding its use for basic research. Given that genome editing for research purposes is indispensable for the realization of clinical genome editing, understanding lay attitudes toward genome editing in research, particularly using human embryos, which is likely to provoke ethical concerns, is helpful for future societal discussion.

**Methods:** An online survey was conducted with Japanese laypeople and researchers to ascertain their views regarding human genome editing for research purposes. Participants were queried about their acceptance as a function of the target of genome editing (germ cells, surplus IVF embryos, research embryos, somatic cells); then, those who answered “acceptable depending on the purpose” were asked about their acceptance in the context of specific research purposes of genome editing. Participants were also asked about their expectations and concerns regarding human genome editing.

**Results:** Replies were obtained from 4,424 laypeople and 98 researchers. Approximately 28.2–36.9% of the laypeople exhibited strong resistance to genome editing for research purposes regardless of their applications. In contrast, 25.5% of the researchers demonstrated resistance only to genome editing in research embryos; this percentage was substantially higher than those concerning the other three targets (5.1–9.2%). Approximately 50.4–63.4% of laypeople who answered “acceptable depending on the purpose” approved germline genome editing for disease research; however, only 39.3–42.8% approved genome editing in basic research to obtain biological knowledge. In contrast, the researchers displayed a lower degree of acceptance of germline genome editing for research purposes related to chronic diseases (60.9–66.7%) than for other research purposes (73.6–90.8%). Analysis of responses concerning expectations and concerns indicated that laypeople who would not accept genome editing of human embryos did not necessarily worry about “instrumentalization of the embryo.” They also had substantially low expectations for recognized advantages of genome editing, including “advances in science” and “reduction of intractable diseases,” compared with other groups of respondents.

**Conclusion:** The assumptions shared among experts in conventional bioethical debates and policy discussions on human genome editing are not self-evident to laypeople.

## 1 Introduction

A Chinese research group reported genome editing in human embryos for research for the first time in 2015 (Liang et al., 2015). CRISPR-Cas9, used in this study, attracted attention because it is a simple and efficient method that accurately edits DNA than previous techniques and is expected to be applied to research and clinical purposes. Although the possibility of genetic modification of human embryos has been previously suggested (Buchanan et al., 2000; Harris, 2007), controversy over the ethics of applying genome editing techniques to reproduction became heated after this Chinese report. Genome editing of human embryos is classified into that performed in basic as well as in clinical research or reproductive medicine. This article focuses on the basic research applications, as injecting human embryos into the uterus after genome editing for clinical is outside its scope.

Genome editing in human embryos for research raises ethical concerns about using human embryos (e.g., NIH, 2015) in research and immediate clinical usage stemming from the blurring of the distinction between basic and clinical research (Lanphier et al., 2015; EGE, 2016). However, some have expressed support for basic research, as genome editing in human embryos may contribute to improving knowledge about human gene function and early embryonic development, as well as advances in research on infertility, genetic diseases, and intractable diseases (Baltimore et al., 2015; ISSCR, 2015; NASEM, 2015).

Despite differing viewpoints, the position of not ethically endorsing the reproductive use of genome editing while its safety or effects remain unclear, and the need to promote public discussion involving various stakeholders, including laypeople, in consideration of the impact of utilization of genome editing on society as a whole, have been emphasized in most statements and reports (Friedmann et al., 2015; ISSCR, 2015; Lanphier et al., 2015; NASEM, 2015; EGE, 2016; NASEM, 2017). In Japan, relevant ministries and agencies partly approved human embryo genome editing for basic research in April 2019 based on discussions held by the Expert Panel on Bioethics, Cabinet Office, since 2015 (MEXT and MHLW, 2019, revised in 2021). The Science Council of Japan stated that promoting public discussion is a crucial issue for the future establishment of regulations (SCJ, 2020).

Under these circumstances, the potential utilization of human genome editing in the future has been surveyed among laypeople and experts, including scientists. Although these surveys have provided valuable findings, the following limitations remain to be addressed: first, few have addressed the degree of acceptance of genome editing for research indispensable for clinical applications; second, few have compared the attitudes of laypeople and experts; and third, multiple viewpoints have been combined in survey questions on genome editing.

The first problem is that, as evident from the study by Delhove et al. (2020), who reviewed studies on human genome editing, among the several surveys concerning human genome editing for clinical purposes, only a few focus on human genome editing for research. Previous surveys investigated the acceptance of supporting particular research with public funding (STAT and Harvard, 2016; Musunuru et al., 2017), as well as opinions on using human embryos in research to improve genome editing technology (Pew Research Center, 2016; Pew Research Center, 2018). However, surveys that directly evaluate the attitudes toward genome editing in research have not been conducted. Musunuru et al. (2017) conducted a survey among physicians and researchers to review their attitudes toward genome editing in germ cells and fertilized eggs for basic research; among the 301 respondents, 68% approved, 22% disapproved, and 10% were uncertain.

As noted earlier, the need for public discussion about the applications of genome editing, especially with respect to editing human germline cells for medical purposes, has been highlighted. However, this does not suggest that sufficient consensus has already been reached concerning the general use of genome editing in research. Basic research involving genome editing of germ cells, embryos, and somatic cells is necessary to realize clinical applications. There are circumstances where positive consideration of the use of research embryos (embryos created exclusively for research), which is generally considered more ethically controversial than the use of surplus IVF embryos (embryos unused after infertility treatment), is unavoidable, depending on its purpose. Therefore, even if respondents support a particular clinical application of genome editing, they may not truly support it if there are serious concerns about human genome editing for research purposes, which is a prerequisite for such use.

The second problem is related to the first problem. Musunuru et al. (2017) only conducted the survey among physicians and researchers; there has been no previous survey in which different stakeholders responded to the same questionnaire at the same time. Therefore, it has been difficult to judge whether responses are characteristic of a particular population or shared among multiple populations. However, investigating the tendency of responses observed in a particular interested party or underlying values may provide guidance for productive public discussions. For example, the continuity from basic research to clinical application is self-evident to experts; however, whether this view is shared by laypeople is uncertain. If the processes leading up to medical care include research using unacceptable methods for counterintuitive purposes as perceived by laypeople, evaluating underlying reasoning is more likely to lead to constructive discussion about the extent to which the technique should be approved. Also, advancing public discussion without such evaluation may end in medicine and science disregarding stakeholder values.

The third problem must be overcome for a comprehensive inquiry into the values of respondents. In previous surveys about human genome editing for clinical purposes, acceptance was occasionally assessed by simultaneously presenting the objective of editing (treatment of disease) and its target (germ cells, embryos); for example, acceptance of genome editing of sperm, egg, and embryos for the therapeutic purposes (Scheufele et al., 2017). At first glance, such a question appears easy to answer intuitively, as it is easy for respondents to imagine the concrete context. However, as it assesses acceptance by combining multiple viewpoints, probing into the reason for selecting the answer from the results is challenging. The answer, “I would not approve,” to the earlier question cannot help conclusively determine whether the respondent opposes genome editing of germ cells or embryos, genome editing to treat disease or both. Therefore, understanding the reasons underlying answers, in addition to a simple yes or no, is required to hold a constructive discussion regarding the ethical/social acceptability of human genome editing.

Considering these problems, we conducted a questionnaire survey to clarify the views of Japanese laypeople about human genome editing for research. The same survey was also conducted among members of the Japanese Society for Genome Editing for comparison. The degree of acceptance of genome editing for research purposes was also investigated using a questionnaire focusing on targets and purposes to obtain responses that more accurately reflect the attitudes of respondents.

## 2 Materials and methods

### 2.1 Survey participants

An online survey was conducted in May 2019. In implementing the survey, we contracted with a private company (GMO Research, Inc.) to develop an online survey platform and collect data. Registered members of the research company’s panel (aged 20–79) and members of the Japanese Society for Genome Editing were recruited to represent the laypeople and researchers, respectively.

The sample size for the lay group was determined using a method employed in prior studies conducted on the Japanese general public (Akatsuka et al., 2021; Sawai et al., 2021). In the prior study, the degree of acceptance of *in vitro* gametogenesis technology was measured using a three-point Likert scale; the data, with a sample size of approximately 3,000, were analyzed using descriptive statistics. This study was also designed to measure the degree of acceptance of genome editing under different circumstances among the respondents using a three-point scale (not acceptable for any purpose, acceptable depending on the purpose, and acceptable for any purpose; details are discussed in the subsequent sections). However, as multiple situations of the use of genome editing could be speculated, the sample size of the general public was assumed to be 4,000 to perform analyses and clarify situation-specific changes in the attitude.

The research company requested for and collected responses from the laypeople registered in their system. These monitor members received email invitations from the research company to participate in our online survey and were free to choose whether or not to participate. The opt-in sampling method (volunteer opt-in panels) was also conducted, and consent to participate in the survey was obtained using a web form (Sue and Ritter, 2007). Thus, it was not possible to track the demographics of non-respondents. Sampling was also performed to ensure that the sex and generation of the respondents remained consistent with the demographics of the general population at the time of the survey (MIC, 2015). The remuneration for this survey is relatively low at JPY29 but is appropriate for online surveys. In addition, the monitors generally can accumulate points, convertible to electronic money, by responding to multiple surveys.

To recruit genome editing experts, a request to complete the survey was sent by email (email intervention) to 335 researchers who were members of the Japanese Society for Genome Editing as of May 2019, with prior permission from the society. As in the case of laypeople, researchers were also sampled using an opt-in method (volunteer opt-in panels) (Sue and Ritter, 2007), and consent to participate in the study was obtained on the website. The researchers did not receive any remuneration for their participation. Two reminder emails were sent to members of the society to increase the response rate.

### 2.2 Determination of the questions

As genetics, including genome editing, is highly specialized and complex to understand for non-experts, prior knowledge about genome editing was expected to affect the responses of the participants. Therefore, the survey was conducted by dividing the general public into groups that were either provided or not provided prior explanation about genome editing. Specifically, basic information about genome editing was presented, and the purposes of human genome editing and differences between somatic and germline genome editing were explained using illustrations.

The data used in this article are part of the “Survey on Human Genome Editing of the Japanese Lay People and Experts project,” and they relate to the following groups of survey items.

1. Questions concerning the level of understanding of science (“literacy score,” see Supplementary Information S1)
2. [For the lay people provided with information only] Explanations on genome editing in general; explanations on human genome editing; questions concerning the level of understanding of the explanations (see Supplementary Informations S2, S3)
3. Questions concerning the expectations/concerns about human genome editing (“expectations/concerns questions”).
4. Questions concerning attitudes to human genome editing for research purposes (“attitudes questions”)
5. Questions concerning the respondents’ demographic characteristics

“What do you expect regarding prenatal and postnatal genome editing?” and “What are you concerned about prenatal and postnatal genome editing?” were prepared as expectation/concern questions. For each question, expectations and concerns concerning human genome editing for research/clinical purposes were listed, and participants were instructed to select all the relevant answers. The expectation/concern questions were prepared by referring to discussions about bioethics and policies (The Nuffield Council on Bioethics, 2016; NASEM, 2017; COB, 2018).

The following nine items were set as expectation questions: “Reduce genetic disorders that are severe and difficult to treat” (Reduction of intractable diseases), “Elucidate mechanisms for diseases of unknown causes” (Clarification of etiology), “Prevent diseases that may affect many people, such as cancer and diabetes” (Prevention of chronic diseases), “Treat life-threatening disease that has limited treatment options” (Treatment of life-threatening diseases), “Advances in science” (Advances in science), “Create genetic diversity to prepare for future crises (e.g., infections, environmental contamination)” (Increase in genetic diversity), “Enhance desirable abilities (e.g., physical abilities, intellectual abilities, appearance) by users” (Enhancement), “Designer babies (babies exactly embodying the parents’ wishes) can be born” (Designer baby), and “Others (free description)” (Others).

The following eleven items were prepared as concern questions: “The tendency to treat sperm and ova as objects is strengthened” (Instrumentalization of gametes), “The tendency to treat fertilized eggs, which are the beginning of human life, as objects is strengthened” (Instrumentalization of embryos), “The occurrence of unintended results in the babies born” (Effects on children), “The occurrence of unintended results in future generations (grandchildren and subsequent generations)” (Effects on future generations), “The occurrence of unintended results in the users of genome editing” (Effects on users), “Clinical application in a stage where the effects on health have not been sufficiently clarified” (Unapproved clinical application), “Social inequality is widened as some people have access to technology and others do not due to economic reasons” (Expansion of social inequality), “Discrimination and prejudice against disabled people is fostered as many people develop a negative view of disability” (Promotion of discrimination), “The genetic diversity of the entire humanity is lost when genome editing is done for short-term benefits” (Loss of genetic diversity), “Use of the technology for the acquisition/improvement of abilities rather than for treatment” (Use of enhancement), “Possibility of the birth of designer babies” (Birth of designer babies), and “Others (free description)” (Others).

All respondents were asked about their attitude toward each genome editing target (questions by target). The specific questions posed were.

• “How do you personally feel about genome editing in sperm and egg?”
• “How do you personally feel about genome editing in fertilized eggs that have not been used for infertility treatment?”
• “How do you personally feel about genome editing in fertilized eggs newly prepared exclusively for research?”
• “How do you personally feel about genome editing in somatic cells?”

The phrase “for research rather than clinical purposes” was appended to all questions. Participants were asked to select one from “acceptable for any purpose,” “acceptable depending on the purpose,” and “not acceptable for any purpose.”

Only those respondents who selected “acceptable depending on the purpose” for each question were presented with specific research purposes and asked to select multiple acceptable purposes (questions by purpose). Five specific research purposes were presented: “Elucidate the causes of infertility and miscarriage, and develop drugs to treat these conditions” (Infertility treatment), “Elucidate the causes of diseases with no radical treatment (e.g., intractable diseases, rare diseases) and develop drugs for their treatment” (Intractable diseases), “Understand the mechanisms of the human being as a living organism and functions of genes” (Basic research), “Elucidate the causes of diseases that affect many people (e.g., cancer, diabetes) and develop drugs for their treatment” (Chronic diseases), and other objectives (Others).

### 2.3 Data analysis

Non-parametric tests (Mann–Whitney U, Wilcoxon signed-rank, and chi-square tests) were performed to assess the results. To clarify the attitude of laypeople resistant to genome editing, they were divided into those who selected “not acceptable for any purpose” and those who selected “acceptable for any purpose” or “acceptable depending on the purpose” to the questions by the target. Kappa coefficients were calculated to examine the consistency of the attitude of each participant. Binomial logistic regression analysis was performed to describe the effects of the attributes, expectations, and concerns of laypeople on their attitudes. For multivariable logistic regression analysis, explanatory variables were entered by the forced-entry method, and the variance inflation factor (VIF) of each explanatory variable was confirmed to be <10 to avoid multicollinearity.

Statistical significance was set at *p* < 0.01 for laypeople, given the large sample size, and *p* < 0.05 for researchers. In nonparametric tests, the effect size was calculated according to Cohen (1988); *r* = 0.10 represented small, *r* = 0.30 medium, and *r* = 0.50 large effects. Analyses were performed using IBM SPSS Regression 27.0 (IBM Corp., NY, United States) and Microsoft Excel for Mac 16.54 (Microsoft Corp., WA, United States).

## 3 Results

### 3.1 Respondent characteristics

We obtained responses from 4,424 laypeople (2,235 with explanation, 2,189 without explanation) and 98 researchers. Table 1 lists the attributes of respondents. Owing to the sampling specifications, the response rate of the lay group could not be calculated, but that of the researcher group was 29.3%. As no significant difference was observed in the responses of laypeople to most of the attitude, expectation, or concern questions with or without explanation, the results are graphically presented with laypeople as a single group. Concerning the scientific understanding (literacy score) of laypeople, no significant difference was observed between those who were provided and not provided an explanation (See Supplementary Table S1).

**TABLE 1:** Respondents’ demographic characteristics.

	Laypeople (*n* = 4,424)		Researchers (*n* = 98)	
	n	%	n	%
Sex				
Male	2,198	49.7	80	81.6
Female	2,226	50.3	18	18.4
Age				
20–29	563	12.7	11	11.2
30–39	727	16.4	27	27.6
40–49	861	19.5	29	29.6
50–59	730	16.5	25	25.5
60–69	877	19.8	5	5.1
70–79	666	15.1	1	1.0
Marital status				
Married	2,951	66.7	70	71.4
Unmarried	1,473	33.3	28	28.6
Presence or absence of children				
Yes	2,390	54.0	58	59.2
No	2,034	46.0	40	40.8
Experience of infertility treatment				
Yes	273	6.2	15	15.3
No	3,784	85.5	76	77.6
I do not know	283	6.4	4	4.1
Undisclosed	84	1.9	3	3.1
Educational background				
Elementary school	2	0.0	0	0.0
Junior high school	131	3.0	0	0.0
High school	1,335	30.2	1	1.0
Technical college	452	10.2	0	0.0
Two-year college	448	10.1	0	0.0
Four-year college	1,878	42.5	12	12.2
Postgraduate studies (master’s degree)	136	3.1	20	20.4
Postgraduate studies (doctorate)	42	0.9	65	66.3
Household income [yen/year]				
Less than two million	451	10.2	1	1.0
Two to four million	1,037	23.4	10	10.2
Four to six million	929	21.0	12	12.2
Six to eight million	572	12.9	20	20.4
Eight to ten million	345	7.8	16	16.3
Over ten million	404	9.1	25	25.5
Undisclosed	686	15.5	14	14.3
Religious affiliation				
Yes	567	12.8	73	74.5
No	3,607	81.5	14	14.3
Undisclosed	250	5.7	11	11.2
What is your present religion?				
Christian	75	1.7	0	0.0
Buddhist	392	8.9	12	12.2
Islam	1	0.0	0	0.0
Shinto	43	1.0	1	1.0
Hindu	0	0.0	0	0.0
Others	21	0.5	1	1.0
Undisclosed	35	0.8	0	0.0
Would you like to take a genetic test that can predict the likelihood of diseases you may get in the future (e.g., cancer, diabetes)?				
Yes	1,396	31.6	56	57.1
No	1,457	32.9	25	25.5
I do not know	1,571	35.5	17	17.3
Have you or a family member had a serious illness (e.g., cancer, diabetes, heart disease, stroke, pneumonia)?				
Yes	1,911	43.2	56	57.1
No	1,968	44.5	33	33.7
I do not know	394	8.9	5	5.1
Undisclosed	151	3.4	4	4.1
Do you have a medical license?				
Yes	―	―	9	9.2
No	―	―	89	90.8
Do you usually conduct research using human samples?				
Yes	―	―	33	33.7
No	―	―	65	66.3

### 3.2 Expectations for human genome editing

**FIGURE 1:**
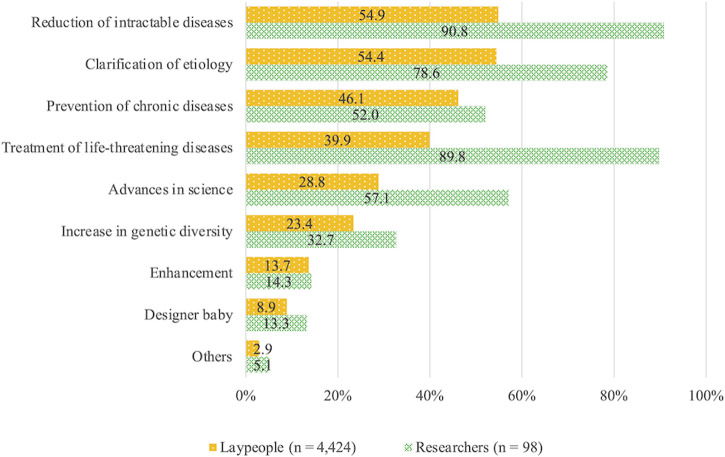
Expectations for human genome editing.

Expectations for human genome editing by laypeople are arranged in descending order (Figure 1). Over half expected reduction of intractable diseases (54.9%) and clarification of etiology (54.4%), whereas less than half expected prevention of chronic diseases (46.1%) and treatment of life-threatening diseases (39.9%).

In contrast, 90.8% of the researchers expressed expectations for decreases in intractable diseases, 89.8% for the treatment of life-threatening diseases, 78.6% for clarification of the etiology, and 57.1% for advances in science; their expectations for all items were substantially higher than those of laypeople. Prevention of chronic diseases, for which the expectation of laypeople was the third highest, was 52.0% in the researchers, which is slightly higher than in laypeople but lower than expectations for items concerning the other diseases mentioned earlier.

Concerning the birth of designer babies (8.9% in the lay group, 13.3% in the researcher group) and use of enhancement (13.7% in the lay group, 14.3% in the researcher group), approximately 10% of both the lay and researcher groups expressed expectations.

### 3.3 Concerns regarding human genome editing

**FIGURE 2:**
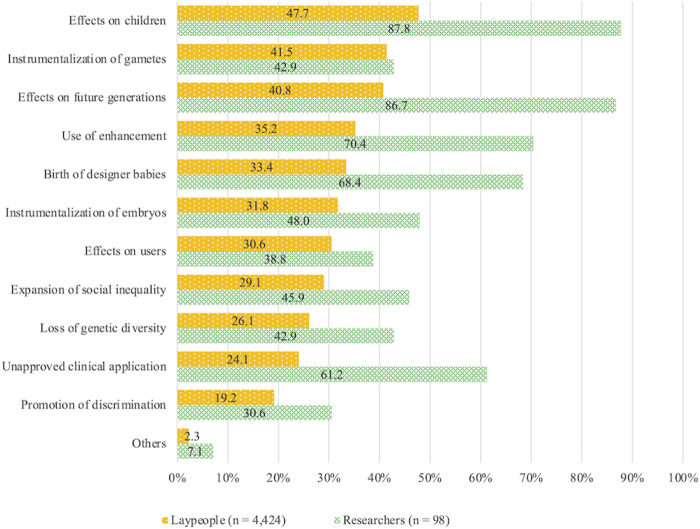
Concerns regarding human genome editing.

The answers to concerns about human genome editing are listed in descending order of the degree of concern in laypeople (Figure 2). The researchers expressed more concerns over all items, except the instrumentalization of gametes than laypeople. While high proportions of both laypeople and researchers expressed concerns over the effects on children (47.7% of the lay group, 87.8% of the researcher group) and effects on future generations (40.8% of the lay group, 86.7% of the researcher group), approximately 90% of the researchers but less than 50% of laypeople were concerned with them. Furthermore, approximately 70% of researchers were concerned over the use of enhancement (70.4%) and the birth of designer babies (68.4%), and 61.2% were concerned over unapproved clinical applications. Conversely, the percentage of laypeople concerned over these items was between 24.1% and 35.2%.

### 3.4 Acceptance according to target

**FIGURE 3:**
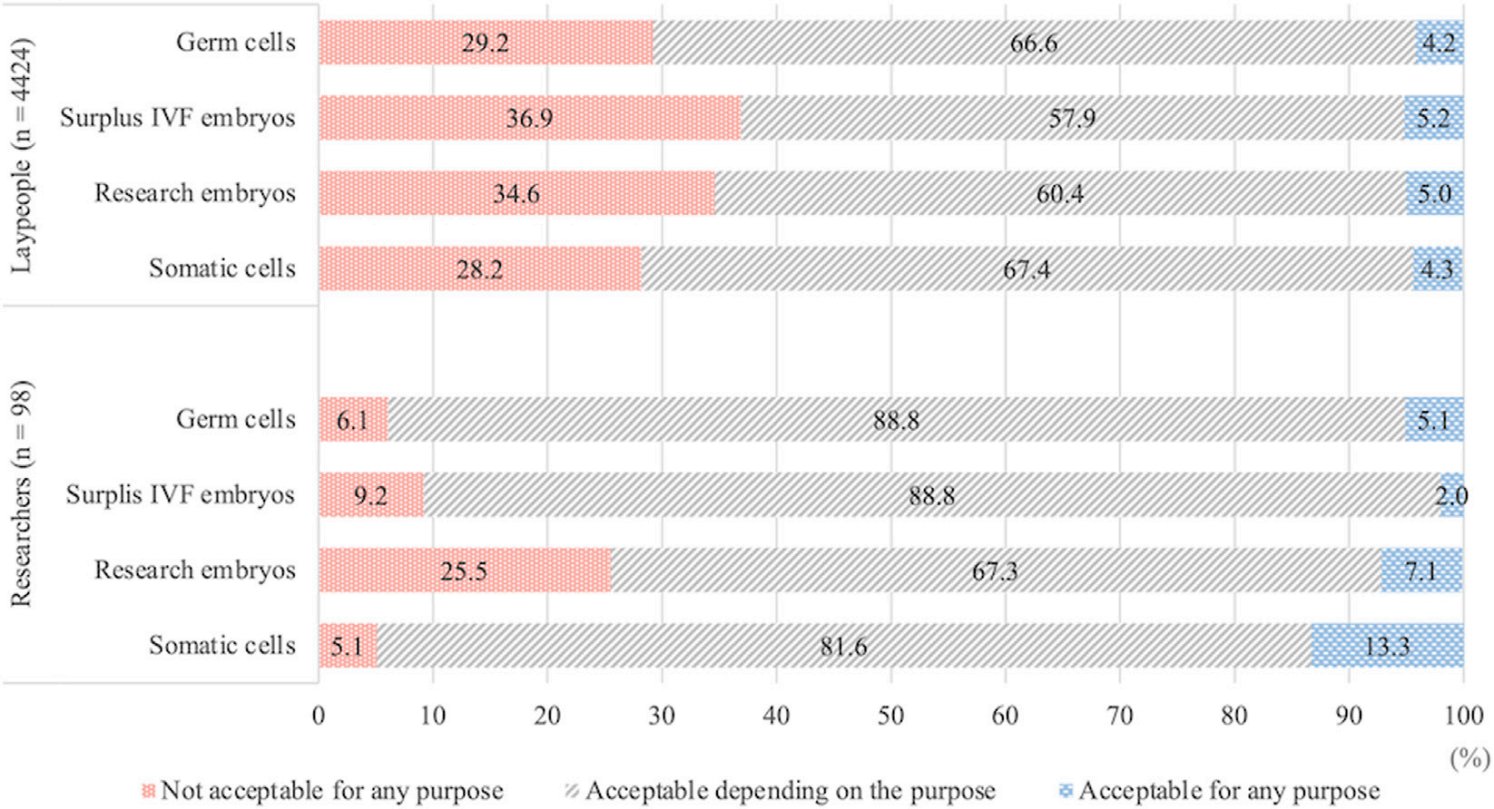
Degree of acceptance of genome editing for research purposes according to target; sums of percentages of all items are not 100%.

The degree of acceptance of genome editing for research in four targets (germ cells, surplus IVF embryos, research embryos, and somatic cells) is displayed in Figure 3. Laypeople answered, “acceptable depending on the purpose,” “not acceptable for any purpose,” and “acceptable for any purpose” in descending order of distribution range in all targets. In contrast, approximately 90% of the researchers selected “acceptable depending on the purpose” for some targets. However, the number of those who chose “acceptable for any purpose” for the application of somatic cells alone was higher than those who chose “not acceptable for any purpose.” The percentage of those who answered that genome editing in various targets was “acceptable depending on the purpose” or “acceptable for any purpose” was 74.5%–94.9% of all researchers and 63.1%–71.8% of all laypeople.

Among the laypeople, 28.2%–36.9% did not approve of genome editing of any target; however, the proportion slightly varied depending on the target. In contrast, the distribution of answers provided by researchers indicated substantial differences, and the percentage of those who answered that genome editing was “not acceptable for any purpose” was 5.1%–9.2% for germ cells, surplus IVF embryos, and somatic cells but 25.5% for research embryos.

We conducted further analysis to comprehensively understand the attitudes of laypeople who selected “not acceptable for any purpose” for genome editing in various targets. Among those who selected “not acceptable for any purpose” for genome editing in multiple targets, moderate consistency was observed when the κ coefficient was calculated to assess the consistency of attitudes to genome editing in each target (0.53 < κ < 0.65, Table 2). Notably, as substantial consistency was observed in attitudes to surplus IVF embryos and research embryos, those laypeople were considered resistant to genome editing in human embryos.

**TABLE 2:** Consistency of the “not acceptable for any purpose” response from laypeople.

	Germ cells	Surplus embryos	Research embryos	Somatic cells
Germ cells				
Surplus embryos	0.62			
Research embryos	0.56	0.65		
Somatic cells	0.58	0.53	0.56	

Subsequently, binominal logistic analysis was performed using the group that chose “not acceptable for any purpose” for both surplus IVF and research embryos and the group that chose “acceptable for any purpose” or “acceptable depending on the purpose” for surplus IVF or research embryos (or both) as objective variables and attributes, expectations, and concerns as explanatory variables. Consequently, multiple regression analysis of the attitude questions and attributes (Cox & Snell *R* ^2^ = 0.10, Table 3) suggested that “age (old),” “married,” and “reluctance to take genetic tests” were significant (*p* < 0.01) among the attributes; these were characteristic of the “not acceptable for any purpose” group.

**TABLE 3:** Demographic characteristics of respondents who do not accept genome editing in embryos for any research purpose

Demographic characteristics	Univariate binomial logistic regression analysis								Multivariate binomial logistic regression analysis^,^ ^b^							
	***B***	***SE B***	***Wald***	***OR***	***95% CI***		***df***	***P***	***B***	***SE B***	***Wald***	***OR***	***95% CI***		***df***	***P***
					***LL***	***UL***							***LL***	***UL***		
Male	−0.31	0.07	21.54	0.73	0.64	0.83	1	0.00	−0.14	0.10	1.88	0.87	0.71	1.06	1	0.17
Age (10-year range)	0.27	0.02	151.00	1.31	1.25	1.36	1	0.00	0.18	0.04	22.46	1.20	1.11	1.30	1	0.00
Literacy Score	−0.02	0.01	3.04	0.98	0.95	1.00	1	0.08	−0.01	0.02	0.12	0.99	0.95	1.04	1	0.74
Educational background	−0.05	0.02	4.73	0.95	0.91	1.00	1	0.03	−0.03	0.04	0.46	0.98	0.91	1.05	1	0.50
Household income	−0.04	0.03	2.15	0.96	0.92	1.01	1	0.14	−0.02	0.04	0.44	0.98	0.91	1.05	1	0.51
Have religious affiliation	0.05	0.10	0.22	1.05	0.86	1.28	1	0.64	−0.18	0.14	1.58	0.83	0.63	1.11	1	0.21
Married	0.70	0.08	81.48	2.01	1.73	2.34	1	0.00	0.43	0.18	5.89	1.54	1.09	2.18	1	0.02
Have child(ren)	0.49	0.07	49.62	1.62	1.42	1.86	1	0.00	0.03	0.15	0.04	1.03	0.77	1.38	1	0.83
Interested in taking genetic testing	−1.15	0.09	172.87	0.32	0.27	0.38	1	0.00	−1.06	0.10	107.39	0.35	0.28	0.42	1	0.00
Have a serious illness	0.13	0.07	3.32	1.14	0.99	1.31	1	0.07	0.04	0.10	0.13	1.04	0.85	1.27	1	0.72
Have undergone infertility treatment	−0.16	0.14	1.26	0.85	0.64	1.13	1	0.26	−0.25	0.20	1.53	0.78	0.53	1.16	1	0.22

B, partial regression coefficient; OR, odds ratio; CI, confidence interval; LL, lower limit; UL, upper limit.

^a^ Among the demographic characteristics, the respondents who chose “Undisclosed” or “I do not know” in the experience of infertility treatment, household income, religious affiliation, genetic testing, and serious diseases were excluded from the analysis.

^b^ Coefficients of determination, Cox-Snell R^2^ = 0.10, Nagelkerke R^2^ = 0.14.

Similarly, multiple regression analysis of attitudes and expectations/concerns (Cox & Snell *R* ^2^ = 0.03, Table 4; Cox & Snell *R* ^2^ = 0.02, Table 5) revealed that the concerns “instrumentalization of gametes,” and “loss of genetic diversity” and the expectations “advances in science,” “increase in genetic diversity,” “reduction of intractable diseases,” and “enhancement” were significant (*p* < 0.01). Thus, the group that considered genome editing in human embryos to be “not acceptable for any purpose” tended to be concerned about the “instrumentalization of gametes,” and “loss of genetic diversity,” as well as not expecting “advances in science,” “increase in genetic diversity,” “reduction of intractable diseases,” and “enhancement.”

**TABLE 4:** Expectations of the respondents who do not accept genome editing in embryos for any research purpose.

Expectations	Univariate binomial logistic regression analysis								Multivariate binomial logistic regression analysis^b^							
	*B*	*SE B*	*Wald*	*OR*	*95% CI*		*df*	*P*	*B*	*SE B*	*Wald*	*OR*	*95% CI*		*df*	*P*
					*LL*	*UL*							*LL*	*UL*		
Advances in science	−0.57	0.08	50.49	0.57	0.49	0.66	1	0.00	−0.62	0.08	55.30	0.54	0.46	0.63	1	0.00
Clarification of the mechanisms of diseases the causes of which are unknown	−0.26	0.07	14.34	0.78	0.68	0.88	1	0.00	−0.13	0.07	3.31	0.88	0.76	1.01	1	0.07
Genetic diversity can be artificially created in preparation for critical situations that the human race will face in the future (e.g., infections, environmental contamination)	−0.66	0.09	55.23	0.52	0.44	0.62	1	0.00	−0.43	0.10	20.14	0.65	0.54	0.79	1	0.00
Genetic diseases that cause severe symptoms and have been difficult to treat (note) can be reduced	−0.33	0.07	23.19	0.72	0.63	0.83	1	0.00	−0.28	0.08	11.03	0.76	0.65	0.89	1	0.00
Diseases that have limited treatments and are likely to be lethal can be treated	−0.23	0.07	11.33	0.79	0.69	0.91	1	0.00	0.03	0.09	0.15	1.03	0.87	1.22	1	0.70
Diseases that may affect many people, such as cancer and diabetes, can be prevented	−0.26	0.07	14.63	0.77	0.68	0.88	1	0.00	−0.11	0.08	1.78	0.90	0.77	1.05	1	0.18
Designer babies (babies exactly embodying the parents’ wishes) can be born	−0.30	0.13	5.59	0.74	0.58	0.95	1	0.02	0.04	0.14	0.08	1.04	0.80	1.36	1	0.77
Abilities desired by the users of genome editing (e.g., physical abilities, intellectual abilities, appearance) can be enhanced	−0.43	0.11	16.39	0.65	0.53	0.80	1	0.00	−0.36	0.11	9.92	0.70	0.56	0.87	1	0.00

B, partial regression coefficient; OR, odds ratio; CI, confidence interval; LL, lower limit; UL, upper limit. a) The coefficients of determination, Cox-Snell *R* ^2^ = 0.03, Nagelkerke *R* ^2^ = 0.04.

**TABLE 5:** Concerns of respondents who do not accept genome editing of embryos for any research purpose.

Concerns	Univariate binomial logistic regression analysis								Multivariate binomial logistic regression analysis^b^							
	*B*	*SE B*	*Wald*	*OR*	*95% CI*		*df*	*P*	*B*	*SE B*	*Wald*	*OR*	*95% CI*		*df*	*P*
					*LL*	*UL*							*LL*	*UL*		
The tendency of treating sperm and ova as objects is intensified	0.44	0.07	41.26	1.55	1.35	1.77	1	0.00	0.34	0.08	20.94	1.41	1.22	1.63	1	0.00
The tendency of treating fertilized eggs, which are the beginning of human life, as objects is intensified	0.33	0.07	21.31	1.39	1.21	1.59	1	0.00	0.13	0.08	2.27	1.13	0.96	1.33	1	0.13
Unintended results may occur in the children to be born	0.08	0.07	1.25	1.08	0.94	1.23	1	0.26	−0.06	0.08	0.53	0.94	0.80	1.11	1	0.47
Unintended results may occur in future generations (grandchildren and subsequent generations)	0.17	0.07	5.96	1.18	1.03	1.35	1	0.01	0.04	0.09	0.17	1.04	0.87	1.24	1	0.68
Unintended results may occur in the users of genome editing	0.22	0.07	9.18	1.24	1.08	1.43	1	0.00	0.10	0.08	1.37	1.10	0.94	1.30	1	0.24
Clinically applied in a stage where the effects on health are not sufficiently clarified	0.18	0.08	5.49	1.20	1.03	1.39	1	0.02	0.05	0.09	0.25	1.05	0.88	1.24	1	0.61
The technology is available to some but not to others for economic reasons, causing expansion of social inequality	0.02	0.07	0.08	1.02	0.88	1.18	1	0.78	−0.19	0.09	4.50	0.83	0.70	0.99	1	0.03
Many people are led to have negative views about disabilities, and discrimination and prejudice against disabled people are promoted	0.21	0.08	6.33	1.23	1.05	1.45	1	0.01	0.07	0.10	0.52	1.07	0.89	1.30	1	0.47
Genome editing for immediate benefits leads to the loss of genetic diversity of the entire human race	0.36	0.07	23.11	1.43	1.24	1.65	1	0.00	0.30	0.09	11.13	1.35	1.13	1.60	1	0.00
Used for the acquisition/improvement of abilities (e.g., physical abilities, intellectual abilities, appearance) rather than for treatment	0.05	0.07	0.53	1.05	0.92	1.21	1	0.47	−0.14	0.09	2.57	0.87	0.73	1.03	1	0.11
May lead to the birth of designer babies (babies exactly embodying the parents’ wishes)	0.12	0.07	2.93	1.13	0.98	1.30	1	0.09	0.03	0.08	0.13	1.03	0.87	1.22	1	0.72

B, partial regression coefficient; OR, odds ratio; CI, confidence interval; LL, lower limit; UL, upper limit. a) The coefficients of determination, Cox-Snell *R* ^2^ = 0.02, Nagelkerke *R* ^2^ = 0.02.

### 3.5 Acceptance according to purpose

**FIGURE 4:**
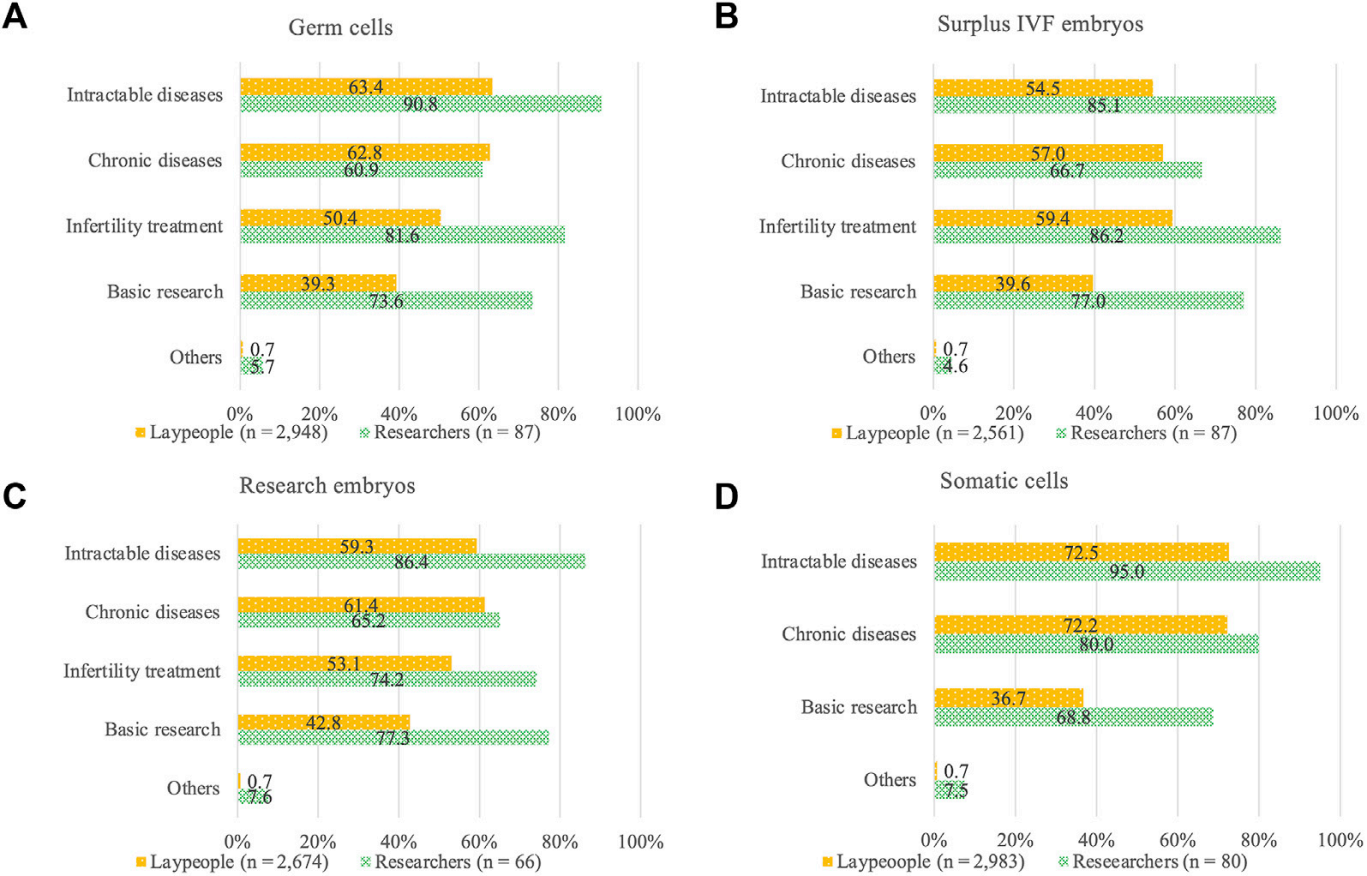
Genome editing for research purposes: degree of acceptance according to purpose. **(A)** Acceptance of research purposes of genome editing in germ cells. **(B)** Acceptance of research purposes of genome editing in surplus IVF embryos. **(C)** Acceptance of research purposes of genome editing in research embryos. **(D)** Acceptance of research purposes of genome editing in somatic cells.

Respondents who selected “acceptable depending on purpose” in the target-specific questions were additionally asked to pick acceptable specific research purposes (Figure 4). Among laypeople, 50.4%–63.4% accepted genome editing in germ cells, surplus IVF embryos, and research embryos for the research on infertility, chronic disease, and intractable disease, but a lower percentage, 39.3%–42.8%, accepted genome editing for “basic research” (Figures 4A–C). In contrast, of the researchers, 73.6%–90.8% accepted genome editing for the research of “infertility treatment” and “intractable diseases” and genome editing for “basic research,” but 60.9%–66.7% accepted genome editing for research concerning “chronic diseases” (Figures 4A–C). Thus, acceptance of research purposes of genome editing in germline cells differed between laypeople and researchers.

A similar response trend between laypeople and researchers was also identified for the acceptance of somatic cell editing (Figure 4D). The proportion of laypeople and experts who accepted somatic genome editing for research on intractable and chronic diseases was approximately 10% higher compared with that of germline cells.

## 4 Discussion

### 4.1 Laypeople, unlike experts, do not perceive differences in embryo types

Based on the target-specific responses, laypeople were more resistant to genome editing in embryos than in germ and somatic cells; however, they showed no difference among types of embryos, surplus IVF versus research embryos. This suggests that lay attitude is affected by whether the target is an embryo but not the embryo type. In contrast, the attitude of the researchers toward genome editing did not substantially differ among the three targets; the proportion of those opposed to genome editing in research embryos alone was markedly higher. This suggests that whether the target is research embryos or not, rather than whether it is an embryo or not, affects the attitude of the researchers.

In Japan, the human embryo has been called the “bud of human life” owing to its potential to develop into a human being. It has been recognized for its unique value, different from somatic cells (CSTP, 2004). Therefore, the use of human embryos for research has been prohibited in principle and approved as an exception only when it has scientific rationale or social validity. Also, as embryos that can be used for research, surplus IVF embryos are supposed to be first, and, in using research embryos (embryos created exclusively for use in research), the rationales of use are more rigidly restricted, based on concerns over instrumentalization of the embryo (CSTP, 2004). The perception that the use of research embryos is more ethically controversial has been shared widely in academic discussions (Devolder, 2015; De Wert et al., 2018).

Ethics seminars for scientists are currently provided at universities and research institutions in Japan; researchers are obliged to attend such a seminar before conducting research in accordance with the national guidelines. The perception of the differences in embryo types acquired through such seminars may underlie the strong resistance to editing in research embryos expressed by researchers. Additionally, researchers are far more likely to be aware that the use of research embryos is more strictly limited than the use of surplus IVF embryos through the guidelines they refer to in conducting research. In contrast, laypeople may not have understood the differences between surplus IVF and research embryos because of their lack of exposure to research guidelines and ethical controversies. Therefore, the different attitudes of laypeople and researchers according to embryo types do not imply that laypeople have lower moral standards than researchers. Some laypeople may think that embryos have specific values, regardless of the purposes for which the embryos were created. This view differs from that of researchers, but it is not to be dismissed and should be regarded as another ethical attitude toward embryos.

Notably, the awareness of the issue based on the differences between surplus IVF and research embryos shared among experts in policy discussions in Japan is not shared by the general public. Our survey data depicts the current scenario wherein society does not sufficiently accept such a distinction. However, we cannot conclude solely from this survey whether this interpretation is generalizable to other countries or a trend unique to Japan. In order to make such a conclusion, it is necessary to conduct similar surveys in countries other than Japan in the future. Moreover, providing the public with basic information, such as types of embryos and their specific research applications, is crucial to discuss future perspectives of genome editing in embryos.

### 4.2 Lay resistance to genome editing in human embryos reflects low expectations

As shown in the degree of acceptance of genome editing by target, 36.9% and 34.6% of laypeople answered that editing in surplus IVF embryos and research embryos, respectively, was not acceptable for any purpose. Also, as indicated by the kappa coefficient, the agreement rate of the proportions of laypeople who considered genome editing not acceptable for any purpose was relatively high between surplus IVF embryos and research embryos. As the explanation document (Supplementary Information S3) clearly stated that human embryos after genome editing for research purposes would not be returned to the uterus, the possibility that the respondents erroneously understood that gene-edited human embryos would be used for reproduction and were concerned about the utilization of the technique is likely to be low. Therefore, a certain proportion of the respondents who chose “not acceptable for any purpose” are likely resistant to genome editing in human embryos in general, regardless of the purpose. However, not all respondents who consistently showed resistance to genome editing in embryos were uniformly opposed to genome editing in somatic or germ cells; they are not considered to have had strong resistance to genome editing for research purposes in general.

The attitudes of respondents who were consistently opposed to genome editing in human embryos can be better understood by the binomial logistic analysis that indicated an association between resistance to genome editing in embryos and low expectations for “advances in science,” “increase in genetic diversity,” “reduction of intractable diseases,” and “enhancement.” According to previous studies about human genome editing for clinical purposes, lay support for its use for enhancement tended to be weak (McCauhey et al., 2016; Scheufele et al., 2017); as anticipated, expectations for enhancement were also low in our survey. However, it is interesting that respondents who disapproved of genome editing in human embryos also had low expectations for advances in science and the reduction of intractable diseases. Advances in science and the treatment of disease are understood as major reasons for supporting genome editing in human embryos and, by experts, as advantages of the utilization of technologies (WHO, 2019). In addition, as such advantages may lead to the survival of people whom the currently available medical treatments cannot save, we speculate that they are relatively acceptable to those resistant to genome editing in human embryos.

However, our survey showed that the advantages of genome editing in human embryos shared by experts were not self-evident to laypeople who express resistance to genome editing in human embryos. In addition, the instrumentalization of the embryo, over which we expected the respondents would be concerned, was unrelated to the disapproval of editing in human embryos. Therefore, it is premature to conclude that the attitude of the respondents who answered that genome editing in human embryos is not acceptable for any purpose was derived from disapproval of using human embryos for research purposes. Instead, we infer that their attitudes are more likely to be explained by their low expectations for advances in science and the reduction of intractable diseases, which have been recognized as advantages of genome editing. While this survey did not reveal it, such low expectations may be due to resistance or suspicion toward the values implicitly presupposed in recent advanced science and technology, such as the eradication of disease and the delay of aging. Although the survey did not directly assess such resistance, it could have been indirectly expressed through low expectations. Therefore, some respondents who opposed the research use of embryos were skeptical about modern science in general rather than having specific concerns about embryonic research.

### 4.3 Laypeople support research with easy-to-understand significance, while experts support research with the rationale of using genome editing

According to the responses to the questions by the purpose, 50.4%–72.5% of laypeople who selected “acceptable depending on the purpose” to target-specific questions were ready to accept genome editing for disease research. In contrast, only 36.7%–42.8% answered that they would accept genome editing in basic research. As this tendency was not limited to embryos but was common to all four targets, including somatic and germ cells, many laypeople who approved of genome editing for research purposes are considered to have distinguished between disease research and research for other purposes and were more receptive to the former.

The tendency of laypeople to approve of disease research was in agreement with their expectations for human genome editing. The top three items about which they had expectations for genome editing, which included the reduction of intractable diseases (54.9%), clarification of etiology (54.4%), and prevention of chronic diseases (46.1%), all disease-related. Conversely, only 28.8% expected advances in science, with a low acceptance of basic research as a purpose of genome editing.

One reason for such tendencies may be that the public supports research with easy-to-understand significance. Since whether the lay respondents were provided an explanation about genome editing or not had little effect on their attitudes, they did not appear to have answered the expectations and attitude questions by reflecting on information about genome editing. The generally low expectations and concerns of laypeople relative to researchers may be explained by the low proportion of lay respondents who sufficiently understood the nature of genome editing as technology and their difficulty in imagining how their expectations and concerns were related to its application. If any laypeople intuitively selected answers, there is no wonder that their expectations focused on items with easy-to-understand significance. In other words, the difficulty in understanding the significance of basic research for laypeople may be the primary reason for their low expectations and acceptance.

In contrast, the researchers may have responded after contemplating whether editing in each target was appropriate or not as the means to achieve specific research goals from a scientific viewpoint. For example, acceptance of genome editing by purpose was generally higher among researchers than among laypeople. However, the proportion of the researchers who accepted research on chronic diseases using germ cells and embryos was 60.9%–66.7%, approximately 20% lower than those who approved of infertility treatment or intractable diseases. Furthermore, the expectations of the researchers for the reduction of intractable diseases (90.8%) and clarification of etiology (78.6%) were high but substantially low for the prevention of chronic diseases (52.0%).

Reasons for the low acceptance and expectations for research on chronic diseases are that cancer and diabetes are included in the category of lifestyle-related diseases rather than genetic diseases and that the technology of genome editing is not likely to lead to clarification of their etiology or discovery of methods for their prevention. The members of the Japanese Society for Genome Editing do not necessarily specialize in disease research. However, professionals with some knowledge in this field may have judged the use of genome editing in the research of chronic diseases to be unreasonable or premature. Furthermore, if researchers selected their answers based on scientific validity, the high acceptance of genome editing in germ cells and embryos for basic research would be understandable. It would be self-evident for most researchers that embryo genome editing in basic research contributes to advances in assisted reproduction technologies, clarification of the etiology of genetic/congenital diseases, and the development of diagnostics and therapeutics.

As observed earlier, our data suggest that the researchers and laypeople selected answers from different viewpoints. Genome editing in human embryos for basic research approved by the Japanese guidelines is also indispensable for disease research but is not sufficiently supported by laypeople. However, such a situation may be attributed to insufficient propagation of its significance rather than any lay objection to basic research. The attitude of laypeople to basic research suggests a lack of appropriate scientific communication about the continuity between disease-related and basic research. To circumvent this issue, information should be presented in a way that better conveys the significance of basic research as the first acceptable purpose of human genome editing for research purposes.

### 4.4 Limitations and significance

As the lay participants in this survey were monitor members of the research company, the attitudes of non-Internet users were not reflected in the results. Therefore, this attribute affected the attitude to genome editing and may have caused bias in the results. This is a limitation of this survey; however, the data could be collected from more than 4,000 laypeople using the Internet. In addition, sampling was conducted according to the demographics of Japan, including the sex and generation of respondents, to minimize bias.

Responses were obtained from members of the Japanese Society for Genome Editing to compare and contrast with those of laypeople. Perceptions of genome editing technology differed between laypeople and experts. However, caution must be exercised while generalizing the responses of the members of the Society for Genome Editing as the attitude of the expert group. Among the 98 researchers who participated in the present survey, 90% did not have a physician license, and approximately 30% routinely used human samples in their research. This suggests they had knowledge about genome editing but did not possess extensive knowledge regarding human diseases. Therefore, different results may have been obtained if our expert group included researchers specializing in genetic diseases or infertility. In this sense, public discussion based on more diverse viewpoints will be improved by expanding the survey participants to researchers with a broader range of specialties.

Despite such limitations, this is a valuable survey investigating the attitudes to genome editing in human embryos for research purposes. Furthermore, the differences between laypeople and researchers in their attitude toward the application of embryos for research and perception about basic research or advances in science clarified by our survey would serve as valuable references in evaluating the prospects of bioscience involving humans, including research on genome editing technology.

## 5 Conclusion

The results of this study can be summarized into three critical points: First, laypeople do not distinguish between surplus IVF and research embryos in the context of genome editing. This indicates that the ethical concerns based on the differences in embryo type shared by experts in conventional discussions are not self-evident to laypeople. In other words, laypeople may evaluate the use of human embryos for genome editing research differently than the researchers. Second, approximately 30% of laypeople who opposed genome editing in human embryos for research purposes tended to have low expectations for advances in science or for overcoming intractable diseases. Based on the lack of strong concerns over the instrumentalization of embryos, their negative attitudes to editing in human embryos may not arise from concerns over the use of embryos for research. Conversely, it may be caused by their low expectations for using embryos in research. Third, while the lay attitudes to human genome editing differed as a function of the perceived significance of the research purpose, the attitudes of the researchers may have differed according to the appropriateness of intervention for particular research purposes. This suggests that the significance of basic research, which is approved prior to clinical application, in biomedical science is not sufficiently understood by laypeople. Our survey clarified that the presumptions shared by experts in policy discussions about human genome editing for research purposes are not self-evident to laypeople. Therefore, providing appropriate information to the general public and seeking ways to make the discussion respect various values are necessary to promote their involvement in societal discussion and include stakeholders with diverse values.

## Data Availability

The raw data supporting the conclusion of this article will be made available by the authors, without undue reservation.
