# Supplementary material for: Genome editing of human embryos for research purposes: Japanese lay and expert attitudes
Source: Front Genet. 2023 Jun 22;14:1205067. doi: 10.3389/fgene.2023.1205067 (PMC10324961; doi:10.3389/fgene.2023.1205067)
Supplement: Supplementary file 1 [file DataSheet2.ZIP › Supplementary_Materials/Supplemental Information 3.pdf]

Supplemental Information 3. Explanations on Genome editing in humans

- Human genome editing has two applications: **research** and **clinical** (Figure 1).
- In **research** applications, genome editing is used to determine the mechanisms of human embryonic development and the functions of genes and to identify the causes of infertility, miscarriage, cancer, and genetic disorders, and to develop medicines to treat these conditions, by using sperm, eggs, fertilized eggs (from which life begins), or somatic cells. Fertilized eggs used for research purposes are usually those that have been left unused after infertility treatment. The use of such unused fertilized eggs alone does not serve the purpose of this research; however, eggs may be fertilized and used specifically for that purpose. *Gene-edited sperm, eggs, fertilized eggs, and somatic cells are never placed back into the human body*.
- **Clinical** applications of genome editing can be divided into two groups according to the purpose of their use: **therapeutic** and **non-therapeutic**. In **therapeutic** applications, genome editing may be used for the radical cure of HIV/AIDS, cancer, intractable diseases, and so on. **Non-therapeutic** uses of genome editing may allow individuals to *acquire new abilities, improve their existing abilities, or prevent diseases*. "*Acquisition of new abilities or improvement of existing abilities*" aims to provide an individual with a feature that he/she does not inherently possess, or improve an ability that he/she already has, such as high physical ability or intelligence, or desirable physical appearance. "Disease prevention" aims to prevent an individual from developing diseases that he/she may develop in the future.

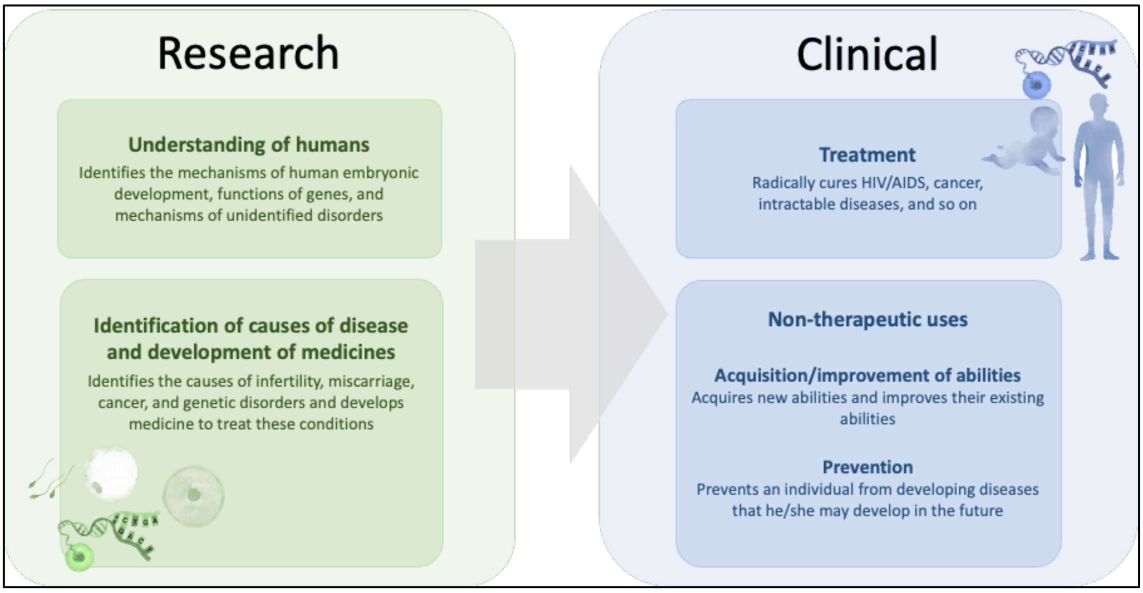

Figure 2. Applications of genome editing in humans

- In clinical applications, genome editing involves placing *gene-edited cells or fertilized eggs* back into the human body. This may occur before or after a person is born (Figure 3).

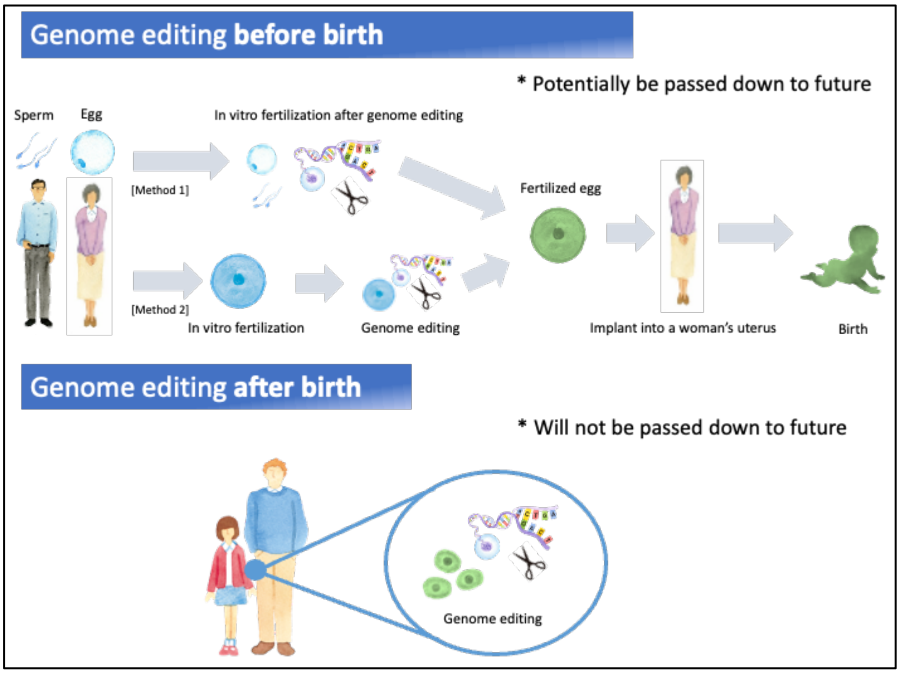

Figure 3. Timing of genome editing in clinical applications

- With genome editing before birth, any change resulting from it can be passed down to future generations, either beneficial or harmful.
- Although the precision of genome editing has been improving, if any DNA that is not targeted is damaged, it may result in unintended consequences. However, not all safety issues have been addressed.

3-1. Did you understand the explanation about "genome editing in humans" above?

**1**<br>I understood it completely	**2**<br>I understood it for the most part	**3**<br>I did not understand it very well	**4**<br>I did not understand it at all
