# Supplementary material for: Genome editing of human embryos for research purposes: Japanese lay and expert attitudes
Source: Front Genet. 2023 Jun 22;14:1205067. doi: 10.3389/fgene.2023.1205067 (PMC10324961; doi:10.3389/fgene.2023.1205067)
Supplement: Supplementary file 1 [file DataSheet2.ZIP › Supplementary_Materials/Supplemental Information 2.pdf]

Supplemental Information 2. Explanations on Genome editing

- Genome editing is a group of technologies that allow scientists to “rewrite” parts of the genome (i.e., the complete set of genetic information in an organism) found in a cell. Specifically, these technologies are used to remove genetic information and/or insert new genetic information (see Figure below).
- All plants and animals, including humans, have genomes that differ from one another. The genomes differ even within the same animal species. Genomes are not static; rather, they may get damaged and repair or repeat the damage.
- While other technologies allow us to rewrite a genome, such as recombinant DNA technology, precise rewriting of target sites is extremely difficult with these technologies. They also require enormous amounts of time and money.
- What sets the latest genome editing technologies apart from the traditional methods is their ability to allow highly targeted rewriting of genomes of many plants and animals, including humans. In addition, it is now possible to create animal or plant models with specific parts of their genome rewritten in a highly efficient manner in terms of time and money if the roles of the DNAs are already known.

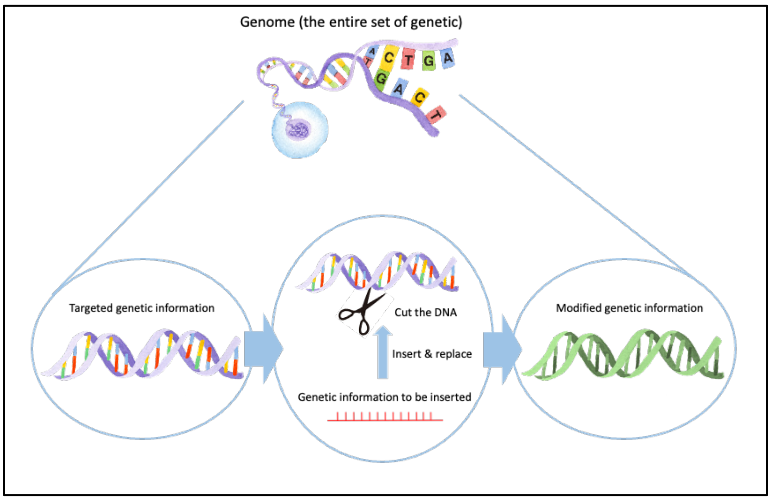

Figure1. How genome editing works

2-1. Were you aware of genome editing?

1<br>I have enough knowledge about it to be able to offer the explanation presented above myself	2<br>I have heard of it	3<br>I did not know about it
