# Supplementary material for: Genome editing of human embryos for research purposes: Japanese lay and expert attitudes
Source: Front Genet. 2023 Jun 22;14:1205067. doi: 10.3389/fgene.2023.1205067 (PMC10324961; doi:10.3389/fgene.2023.1205067)
Supplement: Supplementary file 1 [file DataSheet2.ZIP › Supplementary_Materials/Supplemental Information 1.pdf]

Supplemental Information 1. Understanding of the science

For each of the following statements, please select the one that comes closest to your understanding.

	**1** Definitely false	**2** Probably false	**3** I don't know	**4** Probably true	**5** Definitely true
1. Over time, human beings have incorporated some DNA of different species of animals and viruses into their own.					
2. Personal behavior or environmental factors do not change human DNA sequences.					
3. Tomatoes do not normally carry any gene, while genome-edited tomatoes do.					
4. Scientists have rewritten over 30 genetic features of commercially available plants so far using genome editing technologies.					
5. Gene-edited crops can be legally grown in all of Europe.					
6. Gene-edited product is currently sold at supermarkets.					
7. Scientists have never used genome editing on humans to date.					
8. In Japan, the government (Cabinet Office) is engaged in an ongoing debate about genome editing.					
9. Human beings have evolved from primitive organisms.					
10. There were reports in 2018 on the birth of babies through genome editing in fertilized eggs.					
