# Supplementary material for: Genome editing of human embryos for research purposes: Japanese lay and expert attitudes
Source: Front Genet. 2023 Jun 22;14:1205067. doi: 10.3389/fgene.2023.1205067 (PMC10324961; doi:10.3389/fgene.2023.1205067)
Supplement: Supplementary file 1 [file DataSheet2.ZIP › Supplementary_Materials/Supplemental Table 1.pdf]

Supplemental Table 1. Scientific understanding of the respondents (Literacy Score)

	Mean	Standard deviation	Minimum	Maximum
General public w/ explanation (n = 2235)	3.05	2.48	0	10
General public w/o explanation (n = 2189)	3.04	2.51	0	10
Researchers (n = 98)	7.56	1.23	3	9

Literacy scores were determined using the method employed by Sheufele et al. (2017), and the number of correct answers for each item was tabulated. E.g., for point number 10. There were reports in 2018 on the birth of babies through genome editing in fertilized eggs," the answers "Probably true" or "Definitely true" were regarded as being correct, and the respondents who chose either of these two were given 1 point, while the answers "Definitely false" and "Probably false" were incorrect, and the respondents who chose either were given 0 points. For all items, the answer "I don't know" was scored as 0 points.

Reference

Scheufele, D. A., Xenos, M. A., Howell, E. L., Rose, K. M., Brossard, D., and Hardy, B. W. (2017). U.S. attitudes on human genome editing. *Science* (New York, N.Y.), 357(6351), 553–554. doi:10.1126/science.aan3708
