# Supplementary material for: Genome editing of human embryos for research purposes: Japanese lay and expert attitudes
Source: Front Genet. 2023 Jun 22;14:1205067. doi: 10.3389/fgene.2023.1205067 (PMC10324961; doi:10.3389/fgene.2023.1205067)
Supplement: Supplementary file 2 [file DataSheet1.DOCX]

Supplementary Material

Genome Editing of Human Embryos for Research Purposes: Japanese Lay and Expert Attitudes

Kyoko Akatsuka, Taichi Hatta, Tsutomu Sawai, Misao Fujita

*** Correspondence:** Misao Fujita: misao-fujita@cira.kyoto-u.ac.jp

# Supplementary Information

Supplementary Information 1. Understanding of the science

Supplementary Information 2. Explanations on Genome editing

Supplementary Information 3. Explanations on Genome editing in humans

# Supplementary Table

Supplementary Table 1. Scientific understanding of the respondents (Literacy Score)
